# Consumers’ Perception of Food and Agriculture Education in Farmers’ Markets in Taiwan

**DOI:** 10.3390/foods11050630

**Published:** 2022-02-22

**Authors:** Chun-Chieh Ma, Hsiao-Ping Chang

**Affiliations:** 1 Department of Public Administration and Management, National University of Tainan, No. 33, Sec. 2, Shu-Lin St., Tainan 70005, Taiwan; ccma@mail.nutn.edu.tw; 2 Department of Health Industry Technology Management, Chung Shan Medical University, No. 110, Sec. 1, Jianguo N. Rd., Taichung City 40201, Taiwan; 3 Department of Medical Management, Chung Shan Medical University Hospital, No. 110, Sec. 1, Jianguo N. Rd., Taichung City 40201, Taiwan

**Keywords:** green product, farmer’s market, food and agricultural education, green perceived value, place attachment, Taiwan

## Abstract

Following the trend of green product consumption, farmers’ markets sell green products and have gradually developed the promotion of environmentally friendly knowledge of food and agricultural education. However, past research on farmers’ markets has rarely been combined with the function of food and agricultural education. Therefore, this study explores the relationship between product knowledge, green perceived value, and purchase intention from the perspective of farmers’ market food and agricultural education. Furthermore, research and investigation were conducted with trust and local attachment as multiplicative adjustment variables. This study adopted structural equation modeling (SEM) to examine the structural relationship among local attachment, product knowledge, green perceived value, purchase intention, and model fit. We distributed 396 surveys at three large farmers’ markets in Taiwan, of which 88.6% were used in this study. The research results showed that product knowledge and green perceived value had a significant positive impact on purchase intention; trust positively affected product knowledge and purchase intention; the multiplication of local attachment and trust also positively impacted product knowledge and purchase intention. Based on the research results of this study, it is suggested that while transferring knowledge, the farmers’ markets can incorporate trust and emotional relationships, which will serve as stimulating factors that can increase consumers’ purchase intentions. Although the findings are local to Taiwan, its characteristics are typical of food and agricultural education, as well as farmers’ markets worldwide, especially in Asia.

## 1. Introduction

In the modern age, when health awareness is rapidly increasing, the issues of environmentally friendly land, healthy eating, and food safety are getting more and more attention than ever. However, in the pursuit of technological advancement and modern development, the public’s understanding of and exposure to agriculture, food, environment, and land culture have been declining nowadays [1,2]. Based on the above problems, the quality and safety of agricultural products have gradually become the public’s primary concern [3], which highlights the importance of food and agricultural education [4].

The rise of planting methods that are more environmentally friendly, with the increase in food materials, have led the public to pursue production methods that are more environmentally friendly, and food materials [3], and the green agricultural products they produce, have indirectly promoted the development of the farmer’s market [1]. Specifically, local farmers deliver fresh, local food to a growing number of shoppers who demand food that is not only healthy, but also environmentally friendly. However, farmers’ markets take sustainability a step further. They also ensure that farmers can earn a living off sustainably grown food while providing an outlet where communities can find and purchase their products. From a perspective, behind the rows of produce, busy vendors, and eager customers, farmers’ markets are a bustling hub of sustainability [5], and there would exist additional ‘value’ that can ‘green add’ to the functionality of green farmers’ markets in four dimensions: physical functionality, economic functionality, intangible functionality, and emotional functionality.

Specifically, on physical functionality, on average, food travels more than 1000 miles from the point of production to the retail store [6]. On the contrary, many farmers’ markets only allow vendors to sell food produced within 200 miles. Some markets only have food grown within 50 miles. In terms of economic functionality, prices for many conventional and organic products were lower at the farmers’ markets than in nearby supermarkets [7]. Other advantages include intangible functionality, leisure value of farmers’ markets and traditional shops, food safety, and ease of purchase. Almost half of the needs sell organically labeled products, and many more sell chemical or pesticide-free labeled products [8]. Regarding emotional functionality, do you feel better in farmers’ markets? Reasons for this could include personal contact, trust, location, etc. Local or regionally sourced produce travels about 27 times less distance than conventionally sourced produce [9].

Based on the four dimensions, Taiwan promoted the development of farmers’ markets. In physical and economic functionalities, the farmers’ market advocates local production and sales, as well as local food [10], which not only offers a sales platform to farmers, but also provides consumers with access to fresh and locally grown agricultural products [11]. In addition, on intangible functionality, in an era when food safety problems emerge endlessly, new consumption patterns also make the farmers’ market a place for the public to relax on weekends, bringing a new wave, and indirectly developing the function of dissemination and education of food and agriculture knowledge [4]. 

Furthermore, in emotional functionality, based on the fact that the farmers’ market is not only a place to trade agricultural products, but also a bridge for communication between producers and consumers, it means, in other words, the acquisition of knowledge, the satisfaction of questions about food materials, and the trust and friendship between them can allow them to obtain more benefits [2]. That is, trust will also affect consumer purchasing relationships [12]. If consumers establish a trust relationship with farmers, it can become an incentive that affects consumers’ purchase intentions and helps consumers maintain a continuous buying behavior. Establishing a friendship and trust relationship between each other through the exchange of product knowledge and experience presumably can open up consumers’ knowledge of agricultural products and local agricultural knowledge in addition to prompting farmers to clearly understand consumers’ food preferences and choosing the food materials they need. Therefore, it is necessary to understand the preferences and needs of consumers in the process of purchasing food materials to increase their intentions of purchasing [13].

However, most of the research on food and agricultural education issues in the past has focused on the promotion of food and agricultural education to primary and middle school students in schools, such as the study of the relationship between cognition and the behavioral tendency of food and agricultural education for primary school seniors [14], the current situation of the promotion of food and agricultural education in national primary schools, the study of the relationship between administrative support and the willingness of teachers to advertise [15], and the discussion of the problems faced by the combination of food and agricultural education with the nutrition lunch in primary schools [16]. It is important to promote food and agricultural education in schools, but because the food materials sold in the farmers’ markets are all produced by the producers or farmers, the producers and farmers have a good grasp of the predevelopment process, whether it is the production process of the food materials, the cultivation methods, cooking, or the knowledge of the food materials, they are even more helpful in promoting food and agricultural education. Therefore, the farmers’ market can reflect the spirit of food and agricultural education more directly and convincingly [17].

In the past, many related studies in the farmers’ market, and the majority of the relevant literature, conducted analyses from the perspective of consumers or producers, such as research on the determinants of consumer attitudes and behavior—with the farmers’ market as an example [18], knowledge of food safety and sociodemographic factors of farmers’ market consumers [19], hidden benefits of the short-term food supply chain—taking Italian farmers’ markets as an example [20], consumers’ purchase intention of agricultural products from the farmers’ market [21], the purchase intentions of farmers’ markets using mobile payment [22], and even the willingness of consumers to purchase and loyalty to farmers’ market products [23]. However, Taiwan’s farmers’ markets that promote food and agricultural education are now emerging gradually, but there are few relevant studies to discuss them from the perspective of food and agricultural education. In addition, according to the previous literature and research, there is quite a lot of research on local attachment and trust, but few research studies of the three-order interaction of the two multiplying each other. Therefore, to fill the gaps in the research on the farmers’ markets, this study took food and agricultural education as the entry point to explore the relationship between consumer knowledge of the product, green perceived value and purchase intention, and used trust and local attachment as a three-way interaction for research and investigation. 

Based on possible food and agricultural education and concepts of the farmers’ market that sells green agricultural products, this study attempted to explore the relationship between product knowledge, green perceived value, and purchase intention from the perspective of food and agriculture education in farmers’ markets, and conducted research and investigations with trust and local attachment as multiplicative moderator variables. For these reasons, the study reviews the literature on farmers’ markets and food and agricultural education, product knowledge and purchase intention, perceived value and green purchase intention, trust in product knowledge and purchase intention, and local attachment to trust, product knowledge, and purchase intention. Additionally, then conducting a questionnaire distribution from three large farmers’ markets in Taiwan. After analyzing the results, this study is expected to determine the perception of consumers about food and agriculture education in farmers’ markets in Taiwan. The results of this study should help promote Taiwan’s food and agricultural education and the achievements of green products, and contribute to the concrete implementation of sustainable environmental development and the transmission of knowledge about food safety.

## 2. Literature Review

### 2.1. Farmers’ Market and Food and Agricultural Education 

“Green products” are based on the principle of not wasting resources [24]. In the process of product production from manufacturing to the finished products, the risk of environmental damage must be minimized, waste and hazardous materials must be reduced, and safe and environmentally friendly resources and energy must be used to achieve the production method of environmental friendliness and environmental protection [25]. Compared with traditional products that do not pay too much attention to regulations, green products focus on saving resources and energy, reducing waste discharge, and having the efficiency of recycling, which can significantly reduce the harm to the environment. It also has advantages for environmental protection and sustainable development [26]. Green agricultural products are rice, fruits and vegetables, meat, fresh aquatic products, eggs and milk, etc., as well as processed agricultural products that meet the conditions and standards of green product specifications. Nowadays, when energy and resources are being wasted and the ecological environment is gradually deteriorating, the emergence and widespread use of green agricultural products can improve the harm of harmful substances to the environment [27]. For this reason, Fraccascia, Giannoccaro, and Albino [28] suggested that any product that can cut down and reduce damage to the environment in the life cycle from its growth to recycling can be called a green product. The green products in this study refer to green agricultural products. It is defined as using environmentally friendly resources and production methods for agricultural products to reduce damage to the environment and achieve the functions of a friendly environment and sustainable development.

The emergence of the farmers’ market is to help farmers have a platform for selling agricultural products. It enables farmers to increase their income and drives the development of agriculture, local communities, as well as economic growth. As it takes into account the environment to achieve sustainable development, it has gradually become a better choice for promoting green products [29]. In addition, the market encourages the public to break away from the previous consumption mode and switch to another way of purchase that is friendly to the environment and land and can make food a safe choice for farmers and consumers as well [1].

Food and agricultural education can be defined as an experiential education activity that cultivates the public’s understanding of the basic agricultural production process and achieves the connection between agriculture, environment, and diet [30]. Via such an experiential education activity, the public’s respect for the land, the environment, the producers, and the food itself is cultivated to achieve the sustainable development of health and land agriculture [31,32,33]. In other words, food and agricultural education not only attaches importance to experiencing agricultural education activities, but also the need to understand environmental education. The public can better experience the production of nature, cultivate cherishment toward the environment, and transform the learned experience and knowledge into their own experience accumulation through their personal experience of agricultural activities and understanding of environmental resources, ecology, and pollution [34].

Promoting food and agricultural education allows people to contact agriculture. Its related industries have shifted their educational background to agricultural education and health, nutrition, and natural resources [35]. In order to implement the spirit of “food and agricultural education” in daily life, the localization of the product and focusing on the locally produced crops must be put into consideration [36]. People should learn about the source of the diet and its nutritional value via getting to know the food materials, learn to cook the food materials, and know the place of production and the producer. They can establish the knowledge of food and agricultural education in the most basic direction and maximize the benefits of food and agricultural education. Accordingly, the deepened relationship between food and the environment, and the closer connection between food and local emotions through food and agricultural education can better promote food safety and the culture of green consumption [37].

In summary, nowadays, when energy and resources are being wasted and the ecological environment is deteriorating, the emergence and widespread use of green products are one of the ways to eliminate the environmental hazards due to harmful substances [38]. To extend the definition of green products, it should be considered as the use of environmentally friendly resources and production methods for agricultural products to reduce damage to the environment, achieve a friendly environment and sustainable development. That is, under the above premises, how to promote the emerging and widespread use of green products is a cause for concern. In the process, there should be more discussions on the functions of farmers’ markets for the promotion and knowledge education of green products [1,2].

### 2.2. Research on Correlation between Product Knowledge and Purchase Intention

Knowledge is one of the key factors that affect consumers during the purchase process [39]. Consumers’ perceived value of a product and its price, quality, product attributes, and social culture are all important factors affecting purchase behavior and intention [40]. As suggested in the research of Chung et al. [41], product knowledge is an important resource for a certain place or area, and the amount of product information will affect the purchase behaviors of individuals and groups, and can be used as the information for purchase decision making [42].

Shih’s [43] research mentioned that consumers will use product-related knowledge for the evaluation of purchasing a certain product. When the externally transmitted and provided product information is more complete and detailed, the consumers’ product knowledge will increase. However, when consumers lack product information and related knowledge, they must seek external professional knowledge to support their purchase decisions to evade the risk of uncertainty [44]. Therefore, the research of Chao [45] further shows that consumers will obtain product-related information and knowledge from their own experience and external information, resulting in a series of intentions and behaviors to purchase products.

Based on the familiarity and understanding of the product, it is easy to generate value and increase confidence and attitude, which in turn affects the purchase intention [46]; on the contrary, those consumers who lack product information will change their acceptance of product information due to the change in their environment [47]. Therefore, the existence of external expertise has a significant impact on consumers with high product knowledge [48]. The research of Kong and Chang [49] also pointed out that when consumers understand product information and have a preference for and valuable perception of the product, the product-related knowledge obtained in the purchase of products will indirectly affect consumers’ purchase intentions.

Based on the aforementioned research, product knowledge has an impact on consumers’ purchase intentions. The more product information and knowledge are provided by external expertise; the more consumers’ product purchase intentions will be stimulated [50,51]. Consumer behaviors and preferences will be affected by the cognition and acquisition of product-related knowledge. Consumers with low product knowledge are more likely to be influenced in their purchasing behaviors due to external recommendations [52]. The level of product knowledge has significant impacts on purchase intention [53]. The research results also show the real understanding of the product knowledge by consumers can promote the purchase intention, that is, product knowledge will have a positive impact on the purchase intention [54]. Therefore, if consumers have sufficient product knowledge, it is more likely to encourage consumers to have a higher purchase intention of products and services [55].

In summary, according to previous scholars’ research and research results, product knowledge usually positively affects consumers’ purchase intentions and behaviors. Therefore, this study inferred that the product knowledge conveyed through the food and agricultural education viewpoints of farmers in the farmers’ market will affect consumers’ purchase intentions. The following hypothesis is inferred from this study:

**Hypothesis 1** **(H1).** *Product knowledge positively and significantly affects consumers’ purchase intention.*

### 2.3. Research on Correlation between Green Perceived Value and Purchase Intention

The research of Petrick [56] considers that the quality and feelings of products and services will have an impact on consumers’ purchase behavior and intention [57]. In other words, when the value of products and services is high, the perceived value will increase, making the purchase intention relatively increased. The perceived value is derived from the judgment of consumers on the product or service, and the cost will become one of the factors that affect the consumers’ perceived value.

Continuing the above, Jamal and Sharifuddin [58] pointed out that if the cost paid is higher, the perceived value will decrease; conversely, if the cost paid is lower, the perceived value will increase. The study by Lai [59] also showed that when the values and feelings perceived by consumers are higher, indicating that the benefits are higher than the cost, it means that the positive value is obtained, thereby affecting the consumer’s purchase intention. Perceived value is one of the most significant factors affecting consumers’ purchase intentions and has a positive impact on consumers’ behaviors and intentions to purchase products [60]. Gan and Wang [61] mentioned that the perceived value has a positive impact on consumers’ purchase behaviors. Patterson and Spreng [62] indicated that consumers’ views explain the generation of perceptual value, and the concept of “green” is added for discussion. The definition of the green perception value is that consumers feel the concept and expectation of environmental sustainability from the products and services they receive or pay for. This study defines the green perception value as the consumer’s subjective feeling that when purchasing green products, they believe that the product does not use pesticides and contributes to the sustainable development of the environment. 

Regarding green perceived value, Yu [63] pointed out that the development of an environmental perspective from a green perspective will affect consumers’ green perceived value and their attitude and willingness to purchase green and environmentally-friendly products. However, Chen’s [64] research mentioned that consumers’ perceived value of green and environmentally-friendly products affects their purchase intentions and helps inspire producers to produce more high-quality products. Therefore, for consumers, the green perceived value is quite important. In addition, Chia’s [65] research results indicated that the green perceived value obtained from green products positively and significantly affects consumers’ purchase intention. It was even revealed by the research results of Han et al. [66] that if the product’s green perceived value can reach consumers’ expectations, it will have a positive impact on consumers’ purchase intentions.

In summary, according to previous studies and research results of scholars, the green perceived value usually positively affects consumers’ purchase intention. Therefore, this study inferred that the green perceived value of consumers in the farmers’ market will affect their purchase intention. Below is the hypothesis inferred by this study:

**Hypothesis 2** **(H2).** *Green perceived value positively and significantly affects consumers’ purchase intention.*

### 2.4. Analysis of Impact of Trust on Product Knowledge and Purchase Intention

Trust is an important factor bridge between the person who is trusted and the person who trusts, while the source of establishing trust is the person who trusts receives the professional knowledge and capacity provided by the person who is trusted [67]. In this regard, the research of Koohang et al. [68] showed that the amount of knowledge helps to increase consumers’ trust in products. In other words, when consumers have confidence in the product brand and the provider of product knowledge, they have a certain degree of understanding of product-related information [69].

The degree of consumers’ trust in a product will affect their choice and intention to purchase a product [70]. In other words, since trust is a consumer’s response of dependence and affirmation to the product and its brand, different products will reflect different relevant information and knowledge, which will also affect consumers’ choices of products. If the understanding and knowledge of the product are enhanced, trust will improve consumers’ uncertainty about the product and increase their degree of trust [71]. Therefore, consumers’ increased trust in the product can have more influence on consumers’ behavior in purchasing the product [72]. Furthermore, past experience and word-of-mouth marketing promote trust in the product, product-related knowledge, and personnel, which will enhance consumers’ purchase intention of the product and brand.

In continuation of the above, the research of Ridings et al. [73] proposed that the existence of trust significantly affects the product knowledge acquired by consumers, and the previous product-related knowledge also affects their own attitudes, thus, affects the trust in the product. For an in-depth discussion, the research results of Deng [74] illustrated that consumers’ product knowledge has a positive impact on their trust. In addition, as shown in the research results of [75,76,77,78] Chen and Barnes, Mainardes and Cardoso, Guo, Yan, and Feng [75,76,77,78], trust has a positive and significant impact on consumers’ willingness to buy products. Furthermore, Chou [79] pointed out that if the damage to consumers’ interests is reduced, the product and product-related knowledge gained can make consumers have a trust relationship, which in turn affects their behavior and willingness to buy products. Finally, according to the research results of Lin [80], the higher the degree of trust that consumers have, the stronger the influence on the relationship between their product knowledge and purchase intention.

In summary, according to previous studies and research results of scholars, trust not only positively affects product knowledge, but also positively affects consumers’ purchase intentions. Therefore, this study inferred that consumers’ trust in the farmers and the agricultural products they have sold will strengthen the relationship between the product knowledge acquired by consumers and the purchase intention. The following hypothesis was inferred by this study:

**Hypothesis 3** **(H3).** *When consumers have a higher sense of trust in farmers of the farmers’ market, it will increase the effect of product knowledge and purchase intention.*

### 2.5. Impact of Local Attachment on Trust, Product Knowledge, and Purchase Intention

According to the research of Kyle and Mowen, and Scannell and Gifford [81,82], local attachment is an emotional connection between people and the environment and place. When an individual has a special emotional relationship and identification with the place and environment, it makes the individual generate more interest and investment in cognition and behavior. Wang [83] pointed out that emotional relationships between individuals and places, specific places, such as product knowledge and cognitive preferences, as well as product beliefs and behaviors will influence each other. In other words, the individual’s emotional connection to a place and a specific place will produce cognitive and emotional behavioral input to the product [81]. Then, Chiu [84] pointed out that the higher degree of consumers’ understanding of the knowledge about the place and products, the more it will affect the emotional connection that it produces.

As seen from the above discussion, local attachment is the cognitive, emotional, and functional connection between consumers and a place, and when local attachment is combined with behavior, emotion will affect the behavior of individuals [85]. However, the degree of local attachment is affected by contextual preferences. At the same time, the amount of previous experience also significantly influences local attachment [86]. In this regard, the level of willingness to consume depends on the perception of the local environment and place. Therefore, the degree of local attachment increases or decreases consumers’ purchase behavior and intention [87].

In addition, the amount of trust also affects the degree of local attachment. When consumers have a higher level of trust in cognition, their emotions and attitudes towards local attachment will also increase [88]. The results of Yen [89] showed that consumers’ perceived involvement affects their emotional relationships. Similarly, the results of Kyle et al. [90] also supported that consumers’ involvement has a significant impact on local attachment. Furthermore, it was found in the results of Chen [91] that the greater the emotional degree of local attachment, the more it strengthens consumers’ purchase behavior and intention. At last, according to Chiu’s [84] research results, knowledge has a positive relationship with local attachment.

In summary, according to previous studies and research results of the scholars, local attachment has a positive impact on product knowledge. Moreover, the amount of emotional relationship strengthens consumers’ purchase intentions and behaviors. In addition, the level of trust can cause interference in local attachment. Therefore, this study inferred whether local attachment will strengthen the relationship between trust and consumer product knowledge and purchase intention. The following hypothesis was inferred from this study:

**Hypothesis 4** **(H4).** *When consumers have more local attachment to the farmers’ market, it will increase the relationship between their trust in the farmers’ market and the consumer’s product knowledge and purchase intention.*

## 3. Research Methods 

### 3.1. Sample and Data Collection

The subjects of the questionnaire (please refer to Appendix A for details) for this study were set to be consumers who go to farmers’ markets to promote food and agriculture education. Therefore, the researcher personally went to three large farmers’ markets in Taiwan to distribute the questionnaires from July to August 2020, and a total of 396 questionnaires were collected. After 45 invalid questionnaires with incomplete responses were removed, there were 351 valid questionnaires and the response rate of valid questionnaires was 88.6%. The demographic description of the survey samples are presented in Table 1, the majority were women (*N* = 280, 79.8%), with a total of 280 women (counting 79.8% of the total samples); in the age distribution, the majority were 41–50 years old (*N* = 104, 29.6%), followed by 51 years old (inclusive) and older (*N* = 88, 25.1%), and 21–30 years old (*N* = 88, 25.1%); in terms of education level, the majority were colleges/junior college (*N* = 216, 61.5%); in the part of marital status, the majority were married and have children (*N* = 176, 50.1%), followed by unmarried people (*N* = 145, 41.3%). In terms of habitual place of shopping (multiple choice), the traditional market was the majority (*N* = 258, 40.7%), followed by the farmers’ markets (*N* = 139, 21.9%); in terms of monthly purchase frequency, the majority was 2–3 times (*N* = 129, 36.8%), followed by 4 times (inclusive) and above (*N* = 96, 26.8%); finally, in terms of the monthly average disposable amount for food, the majority was NTD 5001–6000 (*N* = 87, 24.8%), with a total of 87 people (approximately 24.8% of total samples), followed by NTD 5000 (inclusive) or less (*N* = 75, 21.4%).

### 3.2. Statistical Analysis

This study used structural equation modeling (SEM) to investigate the structural relationships between local attachment, product knowledge, green perception value, purchasing intentions, and model adaptation. SEM is a method of testing and improving practical models that enables the testing of theoretical models and can explain the causal relationship between variables in a model based on statistical dependence hypotheses. SPSS 22 (IBM, Armonk, NY, USA) analysis is used for descriptive statistics, reliability analysis, and hierarchical regression, and Amos 22 is used to analyze test factors and to model structural equations.

### 3.3. Questionnaire Design

**Figure 1:**
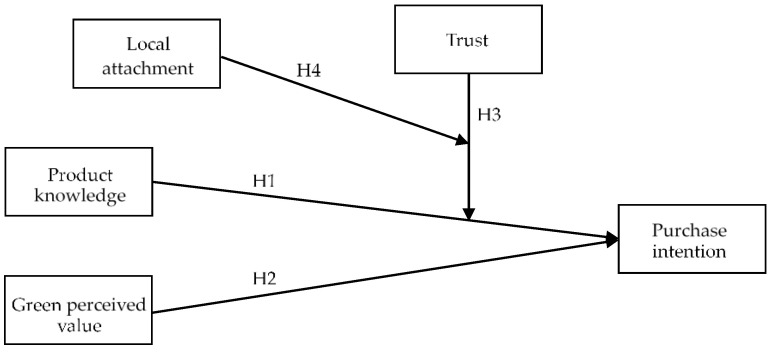
Research framework.

Figure 1 presents a research framework to understand the relationships between product knowledge, green perceived value, purchase intention, local attachment, and trust to promote food and agricultural education on consumer purchase intention in Taiwanese farmers’ markets based on literature reviews. In this framework, the independent variables are product knowledge and green values, purchase intentions are dependent variables, and local engagement and trust are modulator variables. The questionnaire elements were designed based on the review of relevant literature and included the use of a Likert scale of five points, and the five represent ‘strongly agreed’ and the one represents “strongly disagree” (for details, see Appendix A). In the product knowledge section, three items were set, including items such as ‘food and agricultural education promoted by farmers who are familiar with their agricultural products help me increase my knowledge of agricultural products’, and was adapted from Lee with a Cronbach’s α of 0.841 [92]. The three-item scale of the survey’s perception value section includes items such as ‘I think buying green products can contribute to the sustainable development of the environment’, and can be adapted from Chia with a Cronbach’s α of 0.902 [65]. The four-item scale in the purchasing intentions included items such as ‘If there are agricultural products sold on the farmers’ market of food and agricultural education, I would like to buy them’, and was adapted from Huang with a Cronbach’s α of 0.894 [47]. The survey’s four-item trust section includes items such as ‘I think that the agricultural products sold by farmers on the farmer’ market are trustworthy‘, and was adapted from Hsiung with a Cronbach’s α of 0.924 [93]. In the local attachment section, the four-item scale includes items such as ‘I think the green products sold in the local farmers’ market are attractive to me’, and was adapted from Pan with a Cronbach’s α of 0.882 [94]. The Cronbach’s α coefficient of these structures exceeded the suggested 0.70 levels. [95], furthermore, each scale is listed in Table 2.

## 4. Analysis of Results

### 4.1. Reliability and Validity

Table 2 shows the results of the reliability and convergence validity analysis of each construction. Stewart and Volt [96] stated that Cronbach’s α coefficient is high at 0.7, while the coefficient is low at 0.30. Cronbach’s alpha for each dimension is greater than 0.80, indicating high reliability [95]. Fornell and Larcker [97] argue that the composite reliability of persistent variables is greater than 0.60. The high CR of the latent variables indicates that the test variable is valid to measure the latent variable. The coefficient of the CR of variables studied ranges from 0.842 to 0.922, showing that the model is well unified internally. The average difference for each factor (AVE) is 0.640–0.763, higher than the recommended 0.5 standards [98]. However, the factor load value (0.57–0.915) is higher than the recommended 0.5 [99]. In Table 3, the average, standard deviation and correlation between the constructions are presented. There has been found to be a significant positive correlation between knowledge about products and intention to purchase. (*r* = 0.640, *p* < 0.01), green perceived value and in green of purchase intention (*r* = 0.472, *p* < 0.01), trust and purchase intention (*r* = 0.503, *p* < 0.01), and local attachment and purchase intention (*r* = 0.440, *p* < 0.01). The results show that consumers understand products more, perceive green values, and trust and support local communities. They are more likely to buy green products because they have more green products. 

### 4.2. Structural Equation Modeling and Empirical Analysis

**Figure 2:**
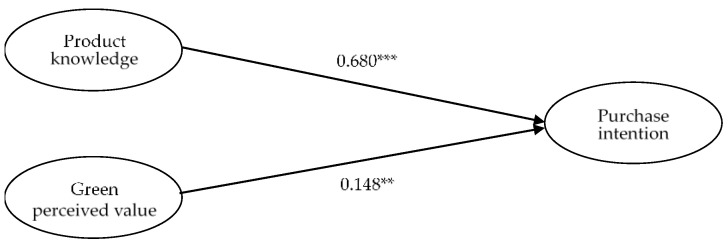
Results of the hypothesized model. Note. ** *p* < 0.01; *** *p* < 0.001.

We used Moment Structure (AMOS) 22 analysis to model structural equations (SEM) and assess path relationships between structures [70]. The results show that the measurement model is good for data (χ^2^/df = 1.318, GFI = 0.976, AGFI = 0.959, CFI = 0.995, NFI = 0.981, IFI = 0.995, RMR = 0.012, RMSEA = 0.030). The ratio χ^2^/df is less than 2 [100], the GFI, the AGFI, the NFI of the CFI and the IFI exceed the recommended threshold of 0.90 [101], and the RMSEA and SRMR values are below the 0.08 separated [102]. This indicates that the data methods used to model the studies are appropriate. Figure 2 and Table 4 show the hypotheses tested from the model data.

Hypothesis H1 was supported. As a result, knowledge of products is strongly and positively related to the intentions of customers to purchase. In the case of the pathway coefficient, 11 is 0.680 (*p* < 0.001). In other words, consumer product knowledge is positive and important with respect to purchase intention; that is, the greater the knowledge of products that consumers acquire, the greater their intention to buy them. Hypothesis H2 was supported. In other words, the perceived value of green is highly positive and related to customers’ purchase intention. The path coefficient 12 is 0.148 (*p* < 0.01). This means that consumers have a positive and important relationship between the value of their green products perceived and purchase intention; the higher the value perceived by consumers in green, the greater their desire to buy.

### 4.3. Testing Moderating Effects

**Figure 3:**
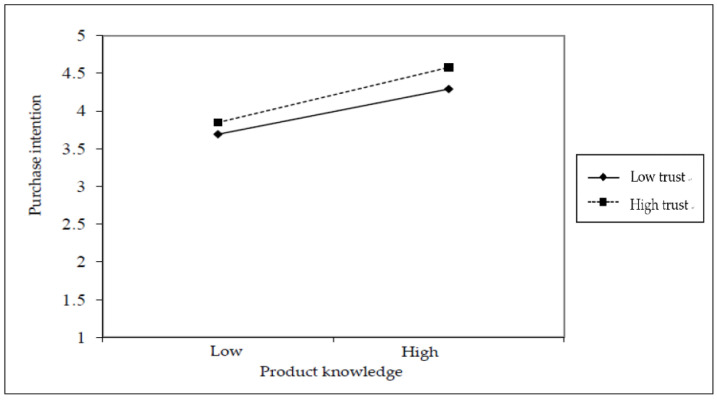
The interactive effect of product knowledge, purchase intention and trust.

Hierarchical regression was used to analyze the moderating effect of trust on the relationship between product knowledge and purchase intention. The analysis results are presented in Table 5. The regression model M1 in this table shows that the value for the effect of product knowledge on purchase intention in the first stage was β = 0.640, *p* < 0.001, the coefficient reached a significant level, and it showed a positively significant correlation. The regression model M2 shows that the value of the impact of trust on purchase intention in the second stage was β = 0.344, *p* < 0.001, the coefficient reached a significant level, and it showed a positively significant correlation. The regression model M3 shows that after adding the interaction value between product knowledge and trust in the third stage, its value was β = 0.093, *p* < 0.05, the coefficient reached a significant level, it showed a positively significant correlation, and the explanatory variation (R^2^) increased to 0.497, the change value of variance explained (∆R^2^) was 0.009, which showed that trust had a moderating effect on the relationship between product knowledge and purchase intention. In addition, based on the results of the hierarchical regression analysis, the values were divided into two parts, low-trust groups and high-trust groups based on the average value of trust, and the effects of these two groups were observed to plot the diagram of the moderating effect of trust on product knowledge and purchase intention, as shown in Figure 3. When consumers had a higher sense of trust in the farmers of farmers’ market, the more the effect between product knowledge and purchase intention was increased; in addition, when consumers had low trust in the farmers of farmers’ market, it also increased the effect between product knowledge and purchase intention. Although both high trust and trust can produce an increased effect compared to low trust, high trust was more effective in predicting the effect between product knowledge and purchase intention. Therefore, Hypothesis H3 was supported. It means that the more trust that consumers have in the farmers of farmers’ markets, the more the effect between product knowledge and purchase intention will be increased.

### 4.4. Testing Three-Way Interaction Effects

This study used product knowledge as the independent variable, purchase intention as the dependent variable, and local attachment and trust as the moderator variables. After the multiplication of the moderator variables, the three-way interaction was adopted to examine whether the multiplication of local attachment and trust have a moderating effect between product knowledge and purchase intention. At the same time, several evaluation criteria were used as the basis for determining the existence of the moderating effect. First, if the coefficient value is positive, it means that the moderating effect is positive moderation; otherwise, it is negative moderation. Furthermore, if the value obtained reaches a significant level and is within the confidence interval of the low limit confidence interval (LLCI) and the upper limit confidence interval (ULCI), and the value 0 is not included, there is a moderating effect; otherwise, there is no moderating effect. 

In this study, the results of the analysis of the effect of the multiplication of local attachment and trust on product knowledge and purchase intention are summarized in Table 6 and Table 7. According to the results of the three-way interaction research, the coefficient value of the multiplication of local attachment and trust on product knowledge and purchase intention was positive, that is, it was a positive moderation. Furthermore, its value was *p* = 0.033, and the change value of variance explained (△R^2^) was 0.006, indicating that the coefficient reached a significant level. In addition, the values of the range in the confidence interval did not include the value 0, indicating that the multiplication of local attachment and trust had a moderating effect on the relationship between product knowledge and purchase intention.

**Figure 4:**
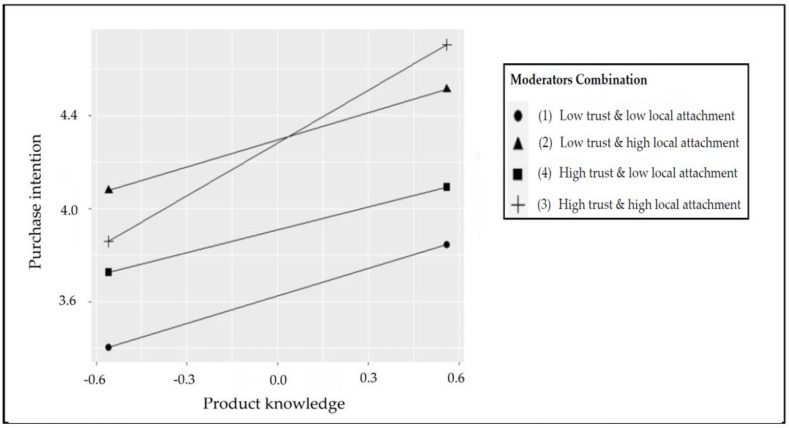
The interactive effects in three directions of local adherence and trust on “product knowledge and purchase intentions”.

However, in order to clarify the type of three-way interaction, it is necessary to verify whether the slope of the hypothetical interaction is significant. As shown in Table 8, regardless of high trust and low trust, the simple slopes are all significant for different levels of local attachment (*b* = 0.32–0.75, *p* < 0.01). In addition, according to the standard of Dawson and Richter [103], a three-way interaction diagram was drawn by this study, as shown in Figure 4. When consumers have high trust in and high local attachment to the farmers’ market, it can better improve the relationship between product knowledge and purchase intention of consumers than low trust and high local attachment. Therefore, as mentioned above, Hypothesis 4 proposed in this study was established.

## 5. Discussion

### 5.1. Relationship between Product Knowledge, Green Perceived Value, and Purchase Intention

According to the analysis of this study, the results showed that product knowledge positively and significantly affected consumers’ purchasing intentions. When farmers in the farmers’ market convey their knowledge of agricultural products from the perspective of food and agricultural education, consumers can deepen their knowledge of agricultural products, thus increasing their purchase intention of the agricultural products. Therefore, the more knowledge of agricultural products transmitted by farmers through food and agricultural education viewpoints that was acquired by consumers, the more likely it was to produce a higher purchase intention. The results of this study are consistent with the research results of Horn and Salvendy, and Chocarro et al. [50,51], that is, it is confirmed that in Taiwan, the knowledge of the product will affect consumers’ purchase intentions, and the higher the knowledge of the product, the more it will stimulate consumers’ purchase intentions for products.

In addition to the above, the results of this study also showed that the perceived value of green positively and significantly affected the intention of consumers to purchase. This result is in line with the research results of Chia [65], who mentioned that the green perceived value that consumers feel from green products will affect the intention to purchase the product of consumers. When consumers feel that the agricultural products sold on the farmer’s market contain the value and benefits that contribute to the sustainable development of the environment, it will increase their green perceived value and increase their willingness to buy agricultural products. Therefore, when consumers’ green value is higher, their purchase intention is higher.

### 5.2. Moderating Effect of Trust on Product Knowledge and Purchase Intention

Based on the research results of Ridings et al. [73], the existence of trust will significantly affect the knowledge about the product acquired by consumers. The higher the trust, the higher the acquired knowledge of the product. According to the analysis of this study, the results also showed that trust had a moderating effect between product knowledge and purchase intention, that is, when consumers had a higher sense of trust in farmers at the farmers’ market, it would strengthen the relationship between product knowledge and purchase intention. In further discussion, the research results of Chen and Barnes, and Mainardes et al. [75,76] all show that trust has a positive effect on consumers’ willingness to buy products. In addition, the research results of Lin [80] mentioned that the higher the degree of trust that consumers have, the more likely it will strongly affect the relationship between product knowledge and purchase intention. It can be seen that the results of this study are similar to the research results of Ridings et al. [73], Chen and Barnes [75]), Mainardes et al. [76], and Lin [80]. That is, if farmers on the farmers’ market give consumers a sense of trust so that consumers feel trust in the farmers and the agricultural products they sell, it can increase consumers’ knowledge of the agricultural products sold on the farmers’ market, and promote their intentions to purchase agricultural products.

In addition to the above, according to the research results of Chiu [84], the higher the consumer’s knowledge of places and products, the more positive emotional connections they will have to place attachment. The results of this study further illustrated that the multiplication of place attachment and trust had a moderating effect between product knowledge and purchase intention, that is, when consumers had a more local attachment to the farmers’ market, it would strengthen the relationship between trust with the knowledge of the product acquired by consumers and their purchase intention. 

Furthermore, according to the research results of Kozak and Rimmington [87], the degree of local attachment increases or decreases the purchase behavior and intention of consumers. Furthermore, Lee and Yun [88] noted that the degree of trust accumulates through the relationship generated by emotions. When consumers have different trust in cognition, their local attachment emotions and attitudes will vary. Therefore, the results of this study also confirmed the results of the research of Kozak and Rimmington [87] and Lee and Yun [88]. That is, when consumers are more attached to the farmer’s market, the more it will affect consumers’ trust in farmers and the agricultural products they sell. The degree to which consumers obtain knowledge about agricultural products will affect the possibility of consumers’ purchase intention of agricultural products.

## 6. Implications, Limitations, and Recommendations

### 6.1. Implications and Contributions

Farmers’ markets are the ultimate green sector of the economy. They are major successes and spurring sustainable economic development. From this study, what additional ‘value’ can ‘green add’ to the functionality of green farmers’ markets would be illustrated in multiple dimensions of functionality. Though there exist ‘functionality’ differences between sales of ‘fresh products’ through traditional shops and farmers’ markets, intangible functionality (leisure food safety, ease of purchase, etc.) and emotional functionality (personal contact, trust, localization, etc.) would play an important role in addition to physical functionality and economic functionality in consumers’ purchase intention in Taiwan. Although the findings are local to Taiwan, its characteristics are typical of food and agricultural education, as well as farmers’ markets worldwide, especially in Asia.

### 6.2. Research Limitations and Recommendations

First, in terms of gender in the samples of this study, there were a larger number of female samples than male samples. However, the previous research and literature on farmers’ markets or purchase intentions for agricultural products show that the proportion of women is relatively high, such as the research of Chung [10] on the impact of product knowledge and quality of relationship on the intention of purchasing in the organic farmer market which shows that the proportion of women is 76.4%. The study by Wang [104] of the impact of market orientation and community customer relationship management on organic small farmers with intention of purchasing as an example shows that the proportion of women is 83%. It is estimated that one of the limitations of this research is that women were the main consumer group in farmers’ markets and for the purchase of agricultural products.

In addition, this study aimed to understand the relationship between the purchase intentions of farmers’ markets consumers and the promotion of food and agricultural education. Only the five items of product knowledge, perceived value of green, purchase intention, trust, and local attachment were adopted as variables to discuss the impact of the promotion of food and agricultural education on the purchase intention of consumers. Of course, there are other more important factors that affect the purchase intention of the consumers of farmers’ markets that promote food and agricultural education, such as experience value. According to the previous research and literature, experience is the important basis for food and agricultural education. So, this is the second limitation of this study. Finally, in terms of research field, this study only conducted research and investigations in farmers’ markets that promote food and agricultural education. However, farmers’ markets are not only classified by promotion of food and agricultural education or not, but also divided into farmers’ markets with a fixed time and place and farmers’ markets of the type of holidays. This is the third limitation of this study.

In view of the limitations of the above-mentioned research, it is recommended that more different factors be included in subsequent research to explore consumers’ purchasing intentions in the farmers’ market after food and agricultural education promotion and their impacts. This is because it cannot only facilitate a better understanding of the factors influencing consumer purchase intentions, but also provide the corresponding suggestions and policies to farmers in the farmers’ market based on the results of the discussion. Finally, and also the most importantly, since the main purpose of the study focuses on consumers’ purchase intention with respect to farmers’ markets, it would likely fail to bridge the intention–behavior gaps. When it comes down to ‘green’, there would be a big difference between what people say (often politically correct) and what people do (more selfish oriented). To address the gaps, research about what actually happens in the buying processes in the markets is strongly urged.

## 7. Conclusions

### 7.1. Promoting Farmers’ Market to Improve Product Knowledge, Green Perceived Value, and Purchase Intention

First, the results of this study showed that the knowledge about the product acquired by consumers in farmers’ markets had a positive impact on the purchasing intentions of consumers. Consumers pay more attention to the knowledge of the product and related information with improved health awareness. Changes in consumer demand make them more willing to choose green products that are environmentally friendly and the ingredients themselves. Furthermore, all green products sold on the farmers’ markets are grown and sold by farmers themselves, so they can provide consumers with valuable information and knowledge about green products. In other words, farmers on the farmer’s market play an important role in acquiring knowledge about green products for consumers. According to the investigation by Mercier [35], the promotion of food and agricultural education can allow the public to be personally exposed to agriculture and its related industries, make the education background not limited to agricultural education, but also cover health, nutrition, and natural environmental resources. Therefore, the farmers’ market should be able to cooperate actively with the central government and local government units to jointly organize special exhibitions of agricultural products to make the public better understand green products and other related food and agricultural experiences. When the farmers’ market provides more information and knowledge about the agricultural products sold, consumers can clearly and fully understand green products, which will further promote their intention to purchase agricultural products. 

Secondly, according to the results of this study, the green perceived value had a positive impact on consumers’ purchase intentions. Based on the fact that green products are important for saving resources and energy, as well as reducing waste emissions, and have recycling efficiency, which can greatly reduce the harm to the environment, they have more advantages for environmental protection and sustainable development [37]. Therefore, when the farmers’ market is more widely established and the sale of green agricultural products is expanded, the perceived value of green by consumers should be further improved. As a result, consumers can think that the agricultural products sold in the farmers’ market are environmentally friendly, and it should increase their willingness to buy agricultural products, which indirectly helps reduce environmental damage and achieve environmental sustainability.

### 7.2. Strengthening Trust to Enhance Agricultural Product Knowledge and Green Product Purchase Intention

Furthermore, more discussions should be held on the functions of green product promotion and knowledge education in farmers’ markets [1,2]. The results of this study showed that trust had a moderating effect between product knowledge and purchase intention. The higher the consumer trust in farmers in the farmers’ market and the agricultural products they sell, the more they will recognize the knowledge and information about the product they propose, and this increases the information related to the product that consumers can obtain and increases the intention of purchasing at the same time. Therefore, farmers in the farmers’ markets are recommended to use social networking sites (such as Facebook and LINE) to establish a friendship and trust relationship with consumers through the dissemination of information, exchanges, and communication. When farmers in the farmers’ market frequently communicate and interact with consumers, consumers will feel a sense of trust. They are also more willing to believe in information about green products delivered by farmers, and thereby increase their purchase intention of agricultural products.

Finally, the results of this study showed that the multiplication of local attachment and trust had a moderating effect between product knowledge and purchase intention. The higher the consumer’s local attachment to the farmers’ market, the more it will affect the level of trust in the farmers of the farmers’ market and the agricultural products sold on the product-related information that consumers can obtain, and thereby increase the purchase intention. Therefore, it is recommended that the farmers’ markets cooperate with local government units to promote food and agricultural education, and promote the farmers’ markets through newspapers, magazines, advertisements, and even through the YouTube platform. In this way, when farmers in a farmers’ market can accumulate consumers’ attachment to the place through more local cohesion, it should also be able to increase consumers’ trust in the product knowledge they have obtained and increase their purchase intention of agricultural products.

## Figures and Tables

**Table 1:** Demographic characteristics.

Variable (*N* = 351)	Category	Frequency	%
Gender	Female	280	79.8%
	Male	71	20.2%
Age	21–30	88	25.1%
	31–40	71	20.2%
	41–50	104	29.6%
	Above 51	88	25.1%
Education	High school	56	15.9%
	University/College	216	61.5%
	Higher education	79	22.5%
Marital status	Single	145	41.3%
	Unmarried with children	10	2.8%
	Married with no children	20	5.7%
	Married with children	176	50.1%
Where to buy agricultural products	Farmers’ market	139	21.9%
	Traditional market	258	40.7%
	Organic shop	92	14.5%
	Supermarket	112	17.7%
	Online shop	33	5.2%
Monthly average purchase frequency	Less than 1 time	64	18.2%
	2–3 times	129	36.8%
	3–4 times	64	18.2%
	Above 4 times	94	26.8%
Monthly average spending on meals	Less than NTD 5000	75	21.4%
	NTD 5001–6000	87	24.8%
	NTD 6001–7000	43	12.3%
	NTD 7001–8000	28	8.0%
	NTD 8001–9000	18	5.1%
	NTD 9001–10,000	41	11.7%
	Above NTD 10,000	59	16.8%

**Table 2:** Results of factor loading, reliability, and validity.

Constructs	Items	Factor Loading	Cronbach’s α	AVE	CR
Product knowledge (PK) [92]	PK1	0.802	0.841	0.640	0.842
	PK2	0.822			
	PK3	0.779			
Green perceived value (GPV) [65]	GPV1	0.849	0.902	0.763	0.906
	GPV2	0.846			
	GPV3	0.915			
Purchase intention (PI) [47]	PI 1	0.798	0.894	0.681	0.895
	PI 2	0.872			
	PI 3	0.853			
	PI 4	0.780			
Trust (TR) [93]	TR1	0.874	0.924	0.748	0.922
	TR2	0.854			
	TR3	0.850			
	TR4	0.895			
Local attachment (LA) [94]	LA1	0.757	0.882	0.681	0.895
	LA2	0.764			
	LA3	0.840			
	LA4	0.867			

CR: Composite reliability; AVE: Average variance extracted.

**Table 3:** Means, standard deviations, and correlations of constructs.

Construct	Mean	S.D.	1	2	3	4	5
1. Product knowledge	4.278	0.559	1.00				
2. Green perceived value	4.297	0.621	0.491 **	1.00			
3. Purchase intention	4.027	0.667	0.640 **	0.472 **	1.00		
4. Trust	4.026	0.645	0.577 **	0.503 **	0.599 **	1.00	
5. Local attachment	3.815	0.690	0.518 **	0.440 **	0.672 **	0.692 **	1.00

Note. *N* = 351; S.D.: Standard deviations; * *p* < 0.05; ** *p* < 0.01; *** *p* < 0.001.

**Table 4:** The model’s standardized regression weights, *t*-values, and hypothesis.

Path	Standardized Regression Weight	*t*-Value	Hypothesis
Directed effect of the integrative model			
Product knowledge ->Purchase intention (γ_11_)	0.608	9.094 ***	H1a *
Green perceived value ->Purchase intention (γ_12_)	0.148	2.660 **	H1b *
χ^2^/df = 1.318, GFI = 0.976, AGFI = 0.959, CFI = 0.995, NFI = 0.981, IFI = 0.995, RMR = 0.012, RMSEA = 0.030			

Note. *t* > 1.96, * *p* < 0.05; *t* > 0.258, ** *p* < 0.01; *t* > 3.29, *** *p* < 0.001; * indicates the hypothesis was supported; GFI: Goodness of fit index; AGFI: Adjusted goodness of fit index; CFI: Comparative fit index; NFI: Normed-fit index; IFI: Incremental fit index; RMR: Root mean square residual; RMSEA: Root mean square error of approximation.

**Table 5:** Results of hierarchical regression analysis.

Variables	Purchase Intention		
	Model 1	Model 2	Model 3
Step 1: Independent variable—Product knowledge	0.640 ***	0.441 ***	0.450 ***
Step 2: Moderator—Trust		0.344 ***	0.334 ***
Step 3: Interaction—Product knowledge x trust			0.093 *
R^2^	0.410	0.489	0.497
∆R^2^	0.410	0.079	0.009
F	242.200 ***	166.294 ***	114.409 ***

* *p* < 0.05; *** *p* < 0.001.

**Table 6:** Three-way interaction model of multiplication of local attachment and trust in product knowledge and purchase intention.

	coeff	se	*t*	*p*	LLCI	ULCI
Constant	−13.078	7.221	−1.811	0.071	−27.281	1.125
Product knowledge	2.894	1.673	1.730	0.085	−0.396	6.184
Trust	4.108	1.866	2.202	0.028	0.438	7.777
Product knowledge × trust	−0.762	0.423	−1.799	0.073	−1.594	0.071
local attachment	4.704	2.055	2.289	0.023	0.662	8.746
Product knowledge × local attachment	−0.818	0.465	−1.762	0.079	−1.732	0.095
Trust × local attachment	−1.256	0.515	−2.440	0.015	−2.269	−0.244
Product knowledge × trust × local attachment	0.244	0.114	2.139	0.033	0.020	0.467

coeff: Coefficient; se: Standard error; LLCI: Lower limit confidence; ULCI: Upper limit confidence interval.

**Table 7:** Three-way interaction test of the multiplication of local attachment and trust in product knowledge and purchase intention.

	ΔR^2^	F	df1	df2	*p*
Product knowledge × trust × local attachment	0.006	4.575	1.000	343.000	0.033 *

* *p* < 0.05.; ΔR^2^: Changes in R-squared; df1: Numerator degrees of freedom; df2: Denominator degrees of freedom.

**Table 8:** Simple slope test.

Items	*b*	SD	*t*-Tests
Low trust and low local attachment	0.39	0.095	4.12 ***
Low trust and high local attachment	0.38	0.132	2.93 **
High trust and low local attachment	0.32	0.136	2.41 *
High trust and high local attachment	0.75	0.108	6.99 ***

SD: Standard deviations * *p* < 0.05; ** *p* < 0.005; *** *p* < 0.001.

## Data Availability

The datasets generated for this study are available on request to the corresponding author.

## Appendix A. Questionnaire

### Appendix A.1. Questions for Purchase Intention, Product Knowledge, Green Perceived Value, Trust, and Local Attachment. All Question Items Are Answered Using a 5-Point Likert Scale (from Strongly Disagree to Strongly Agree)

**Purchase Intention**	**1**	**2**	**3**	**4**	**5**
If there are agricultural products sold at the farmers’ market of food and agricultural education, I would like to buy them. (PI1)	□	□	□	□	□
If there are agricultural products sold at the farmers’ market of food and agricultural education, I would be more interested in buying them. (PI2)	□	□	□	□	□
If there are agricultural products sold at the farmers’ market of food and agricultural education, I would like to continue buying them. (PI3)	□	□	□	□	□
If there are agricultural products sold at the farmers’ market of food and agricultural education, I would like to recommend my relatives and friends to buy them. (PI4)	□	□	□	□	□
**Product knowledge**					
Farmers who are familiar with their own agricultural products are trustworthy for their promotion of the knowledge of agricultural products in the food and agricultural education. (PK1)	□	□	□	□	□
The food and agricultural education promoted by the farmers who are familiar with their own agricultural products help me increase my knowledge of agricultural products. (PK2)	□	□	□	□	□
The knowledge about agricultural products obtained from the farmers who promote food and agricultural education helps me understand the difference between their agricultural products and general products. (PK3)	□	□	□	□	□
**Green perceived value**					
I think buying green products can promote the sustainable development of the environment and will meet my expectations. (GPV1)	□	□	□	□	□
I think buying green products can reduce environmental pollution. (GPV2)	□	□	□	□	□
I think buying green products can contribute to the sustainable development of the environment. (GPV3)	□	□	□	□	□
**Trust**					
I think the agricultural products sold by farmers in the farmers’ market are trustworthy. (TR1)	□	□	□	□	□
I think the agricultural products sold by farmers in the farmers’ market can be eaten with peace of mind. (TR2)	□	□	□	□	□
I think the agricultural products sold by farmers in the farmers’ market are of high quality. (TR3)	□	□	□	□	□
I think the farmers of the farmers’ market attach great importance to consumers’ food safety. (TR4)	□	□	□	□	□
**Local attachment**					
I think the local farmers’ market is of special significance to me. (LA1)	□	□	□	□	□
The green products sold in local farmers’ markets are more satisfying to me than those sold in other places (e.g., supermarkets, mass merchandisers). (LA2)	□	□	□	□	□
I would like to recommend the local farmers’ market to my relatives and friends. (LA3)	□	□	□	□	□
I think the green products sold in the local farmers’ market are attractive to me. (LA4)	□	□	□	□	□

### Appendix A.2. Demographic Profiles

Please answer the following questions based on your actual situation

1. Gender

□ Female

□ Male

2. Age

□ Less than 20

□ 21–30

□ 31–40

□ 41–50

□ 51–60

□ Above 61

3. Education

□ High School

□ University/College

□ Higher Education

4. Marital status

□ Single

□ Unmarried with children

□ Married with no children

□ Married with children

5. Where do you usually buy agricultural products (multiple choice)

□ Farmers’ markets

□ Traditional markets

□ Organic shop

□ Supermarket

□ Online shop

6. Monthly average purchase frequency of agricultural products

□ Less than 1 time

□ 2–3 times

□ 3–4 times

□ Above 4 times

7. Monthly average spending on meals

□ Less than NTD 5000

□ NTD 5001–6000

□ NTD 6001–7000

□ NTD 7001–8000

□ NTD 8001–9000

□ NTD 9001–10,000

□ Above NTD 10,000
